# Clinical and Prognostic Implications of Isocitrate Dehydrogenase Mutation in Gliomas Within a Latin American Public Oncology System: Insights From a Retrospective Panamanian Cohort

**DOI:** 10.7759/cureus.99171

**Published:** 2025-12-13

**Authors:** Francisco Palma-García, Alfredo Abrego, Allyson O´Neill, Raul Lorenzo-Luaces, Maria Salgado, Patricia Chong, Jose Castillo, Rafael E Arauz

**Affiliations:** 1 Department of Medicine, Hospital Santo Tomas, Panama City, PAN; 2 Department of Medicine, Hospital Irma de Lourdes Tzanetatos, Panama City, PAN; 3 Department of Medicine, Ciudad de la Salud, Panama City, PAN; 4 Department of Radiation Oncology, Instituto Oncologico Nacional, Panama City, PAN

**Keywords:** isocitrate dehydrogenase, lower middle income country, oncological prognosis, overall survival (os), glioma

## Abstract

Introduction

Despite the recognized prognostic value of isocitrate dehydrogenase (IDH) mutations in gliomas, real-world survival data from low- and middle-income countries (LMICs), particularly in Latin America, are scarce. This study aimed to evaluate the prognostic impact of IDH mutation status on overall survival (OS) in a national cohort from Panama.

Materials and methods

We retrospectively analyzed 196 glioma patients diagnosed between 2018 and 2023 at the National Oncology Institute of Panama. Tumor histology and grade were classified according to the 2021 WHO Classification. IDH mutation status was assessed via immunohistochemistry. Patients with ependymoma or incomplete clinical records were excluded. Epidemiological variables, treatment modalities, and tumoral and molecular characteristics were collected. Kaplan-Meier survival curves, log-rank tests, and Cox regression models were applied. Analyses were performed using IBM SPSS Statistics for Windows, Version 30 (Released 2024; IBM Corp., Armonk, New York). The study protocol was approved by the Bioethics Committee of The Panama Clinic (EC-CBITPC-063).

Results

Among 196 patients, 59 (30%) were IDH-mutant and 137 (70%) were IDH-wildtype. Median OS was not reached for the IDH-mutant group but was 11.4 months (95% CI: 8.3-14.5) in the IDH-wildtype group (p < 0.001). A total of 123 patients (62.8%) died during follow-up. In multivariable analysis, age ≥ 65 years (HR 1.76; p = 0.008), biopsy-only procedure (HR 2.04; p = 0.004), and Karnofsky Index < 70% (HR 2.02; p = 0.010) predicted worse OS. IDH mutation (HR 0.25; p < 0.001) and frontal-lobe location (HR 0.61; p = 0.039) were associated with improved survival.

Conclusion

In this cohort, IDH mutation emerged as a strong independent predictor of OS. These findings support the routine implementation of IDH testing and the expansion of access to molecular diagnostics in glioma care across LMICs. Prospective studies are warranted to validate these results.

## Introduction

Gliomas are a heterogeneous group of central nervous system tumors originating from glial cells and represent the most prevalent type of primary tumor in the brain and spinal cord. Gliomas account for approximately 31% of all brain tumors and 81% of all malignant brain tumors [1]. In 2021, the World Health Organization (WHO) introduced a classification system for diffuse gliomas occurring in adults, based on the presence of mutations in the isocitrate dehydrogenase (IDH) gene. This classification primarily divides diffuse gliomas into three categories: IDH-mutant (IDH-m) astrocytoma, IDH-m and 1p19q codeleted oligodendroglioma, and IDH-wildtype (IDH-wt) glioblastoma [2].

Mutations in the IDH enzyme occur in a high percentage of low-grade gliomas. Recent studies have demonstrated that mutations in the IDH enzyme influence both the prognosis and treatment response of patients with gliomas [3]. IDH is an enzyme involved in various metabolic processes and exists in three main isoforms: IDH1, IDH2, and IDH3. These mutations alter the enzyme's function in the Krebs cycle, leading to the production of 2-hydroxyglutarate and a decrease in NADPH production (3). Gliomas with IDH mutations, particularly in IDH1 and IDH2, exhibit longer overall survival (OS) compared to gliomas lacking these mutations, also referred to as IDH-wt. This is attributed to the fact that IDH-m gliomas tend to be less aggressive and respond more favorably to treatment [3-5].

Although the prognostic significance of IDH mutation is well established in high-income settings, data from low- and middle-income countries (LMICs) remain scarce.

The primary objective of this study is to compare OS between IDH-m and IDH-wt gliomas within the context of a Latin American public health system. A secondary aim is to assess clinical, surgical, and treatment factors associated with survival outcomes.

## Materials and methods

An observational, analytical, and retrospective cohort study was conducted to evaluate the prognostic impact of IDH mutation status on OS among patients with gliomas. The study was performed at the National Oncology Institute of Panama; medical records were reviewed for cases diagnosed between January 2018 and December 2023. This study adhered to the ethical principles outlined in the Declaration of Helsinki and was approved by the Institutional Bioethics Committee of The Panama Clinic (approval code: EC-CBITPC-063).

The study included all patients aged 18 years or older with histopathologically confirmed gliomas who underwent biopsy or surgical resection and had available IDH molecular analysis by immunohistochemistry (IDH1 R132H). In cases where immunohistochemistry was negative or inconclusive, IDH1/IDH2 mutational analysis was performed using Sanger sequencing to detect non-R132H IDH1 variants or mutations in IDH2, and 1p/19q codeletion status was evaluated using dual-color fluorescence in situ hybridization (FISH). A non-probabilistic consecutive sampling approach was used to include all eligible cases within the study period. Exclusion criteria comprised incomplete medical records, absence of IDH testing, and diagnosis of ependymoma or other non-glioma central nervous system tumors.

Demographic, clinical, radiological, histopathological, molecular, and therapeutic data were extracted from the institutional electronic medical records. Collected variables included age, sex, weight, smoking history, Karnofsky Performance Status (KPS), seizure presentation, tumor location and size, histological subtype and grade according to the 2021 WHO Classification of Central Nervous System Tumors, IDH mutation status (IDH1 or IDH2), presence of 1p/19q codeletion, type of surgical procedure, and adjuvant treatment modalities. Patients were classified into two molecular groups according to IDH status: IDH-m and IDH-wt. Histological categories included astrocytoma, IDH-m (grades 2-4), oligodendroglioma, IDH-m and 1p/19q-codeleted (grades 2-3), and glioblastoma, IDH-wt (grade 4).

The primary outcome was OS, defined as the time in months between the date of histopathological diagnosis and the date of death from any cause or last clinical follow-up. Patients alive at the end of follow-up were censored at their last contact date.

All statistical analyses were performed using IBM SPSS Statistics for Windows, Version 30 (Released 2024; IBM Corp., Armonk, New York). Categorical variables were expressed as absolute frequencies and percentages, while continuous variables were summarized using means, standard deviations, medians, and interquartile ranges. Comparisons between IDH-m and IDH-wt groups were assessed using the chi-square test or Fisher's exact test for categorical variables. OS was estimated using the Kaplan-Meier method, and differences between survival curves were evaluated with the log-rank test (χ²). Median survival and corresponding 95% confidence intervals (CI) were reported. Univariate and multivariate Cox proportional hazards regression models were used to identify independent prognostic factors associated with OS, expressed as hazard ratios (HR) with 95% CI. Variables with a p-value < 0.05 in the univariate model were entered into the multivariate model. Statistical significance was defined as p < 0.05.

## Results

General characteristics

A total of 196 patients with a confirmed histopathological diagnosis of glioma treated at the National Oncology Institute between 2018 and 2023 were analyzed, with various demographic, clinical, and molecular characteristics assessed (Table 1).

**Table 1:** Baseline demographic, clinical, and functional characteristics of patients with IDH-mutant and IDH-wildtype gliomas Comparisons between the IDH-mutant and IDH-wildtype glioma groups were performed using chi-square (χ²). Statistical significance was defined as p < 0.05 (* indicates statistical significance).

Variables	IDH-mutant n=59 (%)	IDH-wildtype n=137 (%)	Total n=196 (%)	X^2^	p-value
Patients' characteristics					
Sex				0.815	0.367
Male	33 (17)	67 (34)	100 (51)		
Female	26 (13)	70 (36)	96 (49)		
Age				9.901	0.002*
>65	6 (3)	43 (22)	49 (25)		
<65	53 (27)	94 (48)	147 (75)		
Weight				0.213	0.644
Weight >70 kg	25 (13)	42 (21)	67 (34)		
Weight <70 kg	19 (10)	38 (19)	57 (29)		
Missing data	14 (7)	57 (29)	71 (36)		
Smoker				1.271	0.259
Yes	10 (5)	14 (7)	24 (12)		
No	39 (20)	91 (46)	130 (66)		
Missing data	10 (5)	32 (16)	42 (21)		
Karnofsky Index				1.244	0.265
Karnofsky <70%	5 (3)	18 (9)	23 (12)		
Karnofsky >70%	53 (27)	106 (54)	159 (81)		
Missing data	1 (1)	13 (7)	14 (7)		
Seizures				21.081	<0.001*
Yes	40 (20)	44 (22)	84 (43)		
No	19 (10)	92 (47)	111 (56)		
Missing data	-	1 (1)	18 (1)		
Tumors characteristics					
Tumor histology and grade				196.000	<0.001*
Astrocytoma, IDH-mutant, grade 2	23 (12)	0	23 (12)		
Astrocytoma, IDH-mutant, grade 3	11 (5)	0	11 (5)		
Astrocytoma, IDH-mutant, grade 4	9 (5)	0	9 (5)		
Oligodendroglioma, IDH-mutant and 1p/19q codeleted, grade 2	10 (5)	0	10 (5)		
Oligodendroglioma, IDH-mutant and 1p/19q codeleted, grade 3	6 (3)	0	6 (3)		
Glioblastoma Multiforme, IDH-wildtype, grade 4	0	137	137 (70)		
Locations (Frontal-parietal vs. others)				3.390	0.656
Frontal	34 (17)	34 (17)	68 (35)		
Parietal	11 (6)	52 (27)	63 (32)		
Temporal	8 (4)	35 (18)	43 (22)		
Occipital	1 (1)	10 (5)	11 (6)		
Other locations	5 (3)	6 (3)	11 (6)		
Tumor size				0.864	0.352
>30 mm	29 (15)	75 (38)	104 (53)		
<30 mm	9 (5)	15 (8)	24 (12)		
Missing data	21 (11)	47 (24)	68 (35)		
Treatment characteristics					
Surgery extension (Biopsy vs. Resection)				2.342	0.125
Only Biopsy	6 (3)	26 (13)	32 (16)		
Partial Resection	32 (16)	74 (38)	106 (54)		
Complete Resection	21 (11)	37 (19)	58 (30)		
Treatment groups (Surgery + RT + CT vs. Others)				8.827	0.003*
Surgery + RT + CT	42 (21)	66 (34)	108 (55)		
Surgery + RT	8 (4)	18 (9)	26 (13)		
Surgery only	3 (2)	27 (14)	30 (15)		
Biopsy + RT + CT	4 (2)	10 (5)	14 (7)		
Biopsy + RT	2 (1)	4 (2)	6 (3)		
Biopsy only	0 (0)	12 (6)	12 (6)		
Patient condition				45.86	<0.001*
Alive	43 (22)	30 (15)	73 (37)		
Dead	16 (8)	107 (55)	123 (63)		

The median age of the cohort was 54 ± 15.72 years (range: 19-88). In the IDH-wt group, the median age was 58 ± 15.73 years (range: 19-88), while in the IDH-m group, it was 45 ± 13.43 years (range: 21-75). Of all patients, 25% (n=49) were over 65 years of age, and 75% (n=147) were under 65. A higher proportion of patients under 65 was observed in the IDH-m group compared to those over 65 (27% vs. 3%). Males accounted for 51% (n=100) and females 49% (n=96). Weight was over 70 kg in 34% (n=67), under 70 kg in 29% (n=57), and not available in 36% (n=71) of the cases (p = 0.644). The KPS was >70% in 81% (n=159) of patients and <70% in 12% (n=23), with no significant differences between groups (p = 0.265). Regarding smoking history, 12% (n=24) reported previous tobacco use, while 66% (n=130) were non-smokers. Seizures at diagnosis were reported in 43% (n=84) of patients, with a higher proportion in the IDH-m group (20% vs. 10% in IDH-wt, p < 0.001). A higher proportion of patients under 65 was observed in the IDH-m group than in those over 65 (27% vs. 3%). Males accounted for 51% (n=100) and females 49% (n=96). Weight was over 70 kg in 34% (n=67), under 70 kg in 29% (n=57), and not available in 36% (n=71) of the cases (p = 0.644). The KPS was >70% in 81% (n=159) of patients and <70% in 12% (n=23), with no significant differences between groups (p = 0.265). Regarding smoking history, 12% (n=24) reported previous tobacco use, while 66% (n=130) were non-smokers. Seizures were reported at diagnosis in 43% (n=84) of patients*.*

Regarding IDH enzyme expression, 30.1% (n=59) of patients had IDH-m tumors, while 69.9% (n=137) were IDH-wt. Among those with IDH mutations, 93% (n=55) had the IDH1 isoform and 7% (n=4) the IDH2 isoform. Histological distribution was as follows: 38 astrocytomas (19.4%), 16 oligodendrogliomas (8.1%), and 137 glioblastomas (69.9%). All astrocytomas were IDH-m, including 23 grade 2 (12%), 11 grade 3 (5%), and nine grade 4 (5%) cases. Similarly, all oligodendrogliomas were IDH-m with 1p/19q codeletion, distributed as ten grade 2 (5%) and six grade 3 (3%) tumors. In contrast, all glioblastomas were IDH-wt (n = 137; 70%), corresponding to WHO grade 4 lesions. Tumor location was the frontal lobe in 35% (n=68), the parietal lobe in 32% (n=63), the temporal lobe in 22% (n=43), the occipital lobe in 6% (n=11), and other locations (thalamus, pineal gland) in 6% (n=11). No significant differences were observed in tumor location (p = 0.656) or tumor size (p = 0.352). The 1p/19q codeletion was reported in 77% of cases with IDH-m oligodendroglioma, with no differences between groups (p = 0.661).

Regarding treatment, 55% (n=108) of patients received multimodal therapy (surgery followed by radiotherapy (RT) with chemotherapy (CT)), which was more common in the IDH-m group (34% vs. 21%, p = 0.002). The most frequent extent of resection was partial (54%, n=106), followed by complete resection (30%, n=58) and biopsy alone (16%, n=32), with no significant differences between groups (p = 0.125).

At the end of follow-up, a total of 37% (n=73) of patients were alive, and 63% (n=123) had died, with a significantly higher proportion of deaths observed in the IDH-wt group compared to the IDH-m group: 55% (n=107) versus 8% (n=16), respectively.

Survival analysis**

**Figure 1:**
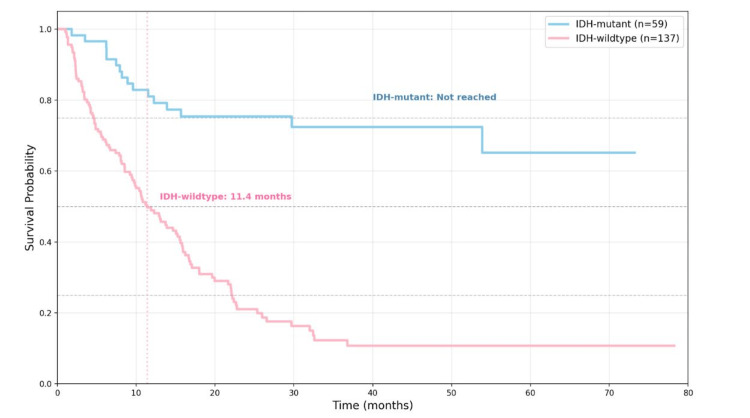
Kaplan-Meier survival curves comparing IDH-mutant and IDH-wildtype patients The log-rank test showed a statistically significant difference between the two groups (χ² = 44.519, df = 1, p < 0.001). Median overall survival was not reached for the IDH-mutant group, whereas it was 11.4 months (95% CI: 8.3–14.5) for the IDH-wildtype group. Statistical significance threshold: p < 0.05.

The median OS for the entire cohort was 15.9 months (95% CI: 12.9-21.7). In the IDH-wt group, median survival was 11.40 months (95% CI: 8.29-14.51), whereas the median was not reached during follow-up in the IDH-m group (Figure 1). In the interquartile analysis, the 25th percentile survival was 22.2 months for IDH-wt and 29.73 months for IDH-m, while the 75th percentile for IDH-wt was 4.6 months. The log-rank test revealed a statistically significant difference between groups (χ² = 44.519, df = 1, p < 0.001), indicating significantly longer OS in IDH-m patients. The median follow-up time was 34.23 months (95% CI: 25.91-42.56) across the cohort, with no significant differences between the IDH-wt (34.23 months, 95% CI: 21.48-46.99) and IDH-m (34.67 months, 95% CI: 23.96-45.37) groups (p = 0.607).

Regarding specific variables (Table 2), patients under 65 years had significantly longer median survival (22.1 months; 95% CI: 15.42-28.78) compared to those aged 65 or older (8.93 months; 95% CI: 7.04-10.83, p < 0.001). No significant survival differences were found by sex (females: 13.1 months; 95% CI: 8.19-18.01, males: 17.07 months; 95% CI: 13.44-20.69, p = 0.316) or weight (≥70 kg: 22.1 months; 95% CI: 18.21-25.99, <70 kg: 15.83 months; 95% CI: 4.32-27.34, p = 0.159)*.*

**Table 2:** Kaplan–Meier overall survival analysis according to clinical, molecular, functional, and treatment-related variables Median overall survival (OS) is expressed in months with 95% confidence intervals (CIs). Survival estimates were calculated using the Kaplan–Meier method, and comparisons between groups were performed using the log-rank (χ²) test. Statistical significance was defined as p < 0.05 (* indicates statistical significance). "Not reached" indicates that fewer than half of the patients in that group experienced the event during follow-up.

Variables	Median overall survival (months) CI 95%	Log-rank (Mantel-Cox) X^2^	p-value
IDH status		44.519	<0.001*
IDH-mutant	Not reached		
IDH-wildtype	11.4 (8.29–14.51)		
Age		21.685	<0.001*
<65 years	22.1 (15.42–28.78)		
≥65 years	8.93 (7.04–10.83)		
Sex		1.004	0.316
Female	13.1 (6.19–18.01)		
Male	17.07 (13.44–20.69)		
Weight		1.983	0.159
≥70 kg	22.1 (18.21–25.99)		
<70 kg	15.83 (4.32–27.34)		
Karnofsky		14.852	<0.001*
≥70%	19.63 (14.75–24.52)		
<70%	7.87 (4.39–11.35)		
Smoking		0.079	0.778
Non-smokers	21.67 (16.37–26.96)		
Smokers	16.23 (12.92–19.55)		
Seizure Presentation		9.618	0.002*
Seizures	25.97 (15.93–36.01)		
No seizures	13.00 (9.76–16.24)		
Location		15.490	0.004*
Frontal lobe	32.6 (8.26–56.94)		
Other locations	13.1 (9.24–16.96)		
Treatment		80.329	<0.001*
Surgery + Adjuvant	22.8 (13.40–22.60)		
Biopsy + Adjuvant	15.7 (14.3–17.1)		

Patients with a Karnofsky index ≥70% had a median survival of 19.63 months (95% CI: 14.75-24.52), compared to 7.87 months (95% CI: 4.39-11.35) for those with a Karnofsky index <70%, showing a significant difference (p < 0.001). Non-smokers had a median survival of 21.67 months (95% CI: 16.37-26.96) versus 16.23 months (95% CI: 12.92-19.55) in smokers, with no significant difference (p = 0.778). Patients presenting with seizures had a median survival of 25.97 months (95% CI: 15.93-36.01), which was greater than that of 13.00 months (95% CI: 9.76-16.24) in those without seizures (p = 0.002).

Patients with glioblastoma, IDH-wt, showed the shortest median survival of 11.4 months (95% CI 9.43-15.27). In contrast, the median survival was not reached for astrocytomas, IDH-m and oligodendrogliomas, IDH-m with 1p/19q codeletion.

**Figure 2:**
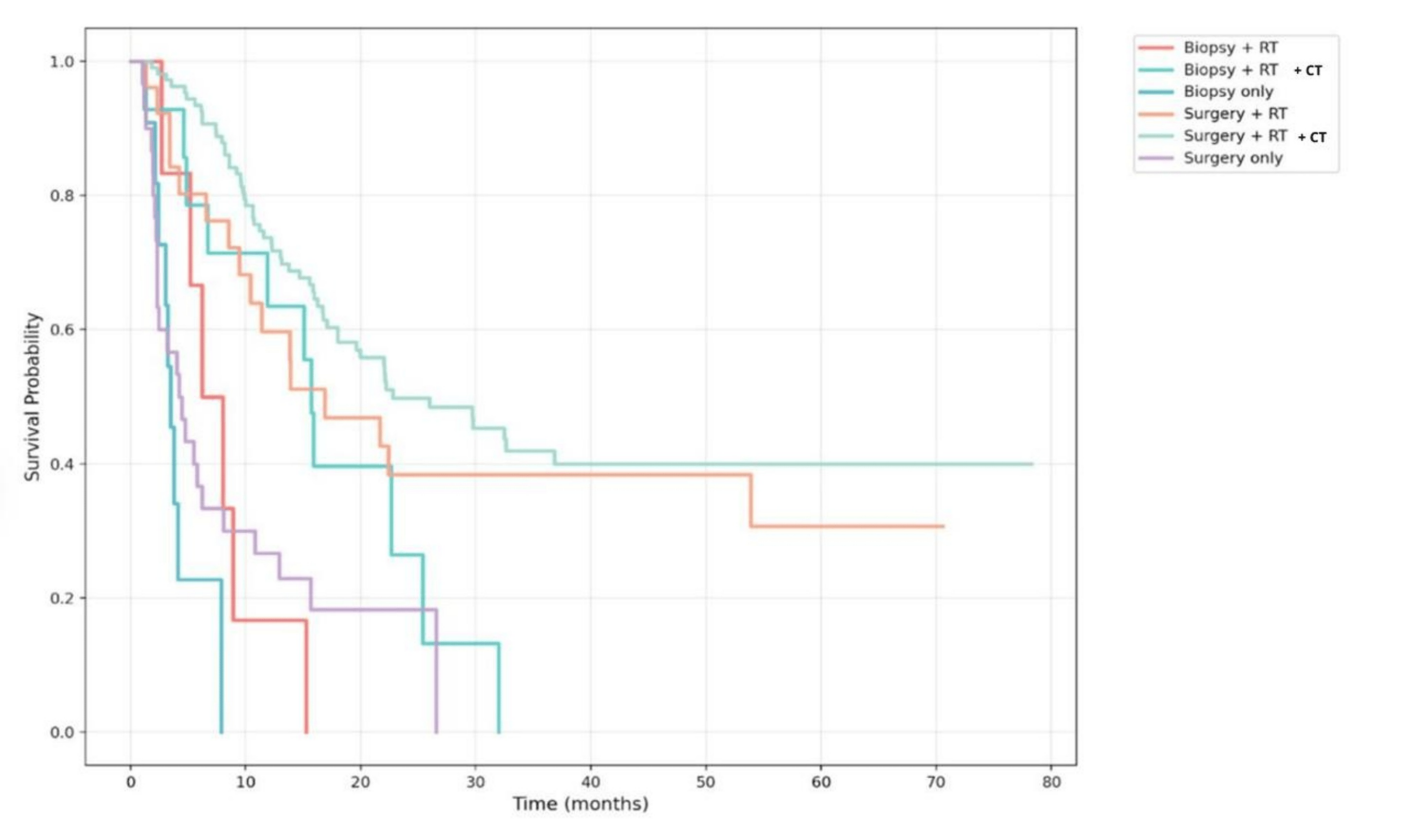
Kaplan–Meier overall survival curves according to treatment modality Patients undergoing surgery followed by adjuvant radiotherapy (RT) and chemotherapy (CT) achieved the best outcomes (median = 22.8 months; 95% CI: 12.5–33.1), whereas those treated with biopsy alone had the poorest survival (median = 3.5 months; 95% CI: 2.8–4.2). Statistical comparison was performed using the log-rank test (χ² = 29.782, df = 4, p < 0.001). Statistical significance threshold: p < 0.05.

Patients who underwent biopsy alone had the lowest survival: 3.5 months (95% CI: 2.8-4.2). Those who received biopsy followed by radiotherapy had 6.2 months (95% CI: 2.8-9.6), and with the addition of chemotherapy, 15.7 months (95% CI: 14.3-17.1). Patients who underwent surgery alone had a median survival of 4.2 months (95% CI: 2.2-6.3), which increased to 16.9 months (95% CI: 4.7-29.0) with radiotherapy and reached the highest value, 22.8 months (95% CI: 12.5-33.1), when chemotherapy was added (Figure 2).

Factors associated with survival

Cox regression analysis (Table 3) revealed that several variables were significantly associated with OS. In the univariate model, age ≥65 years (HR: 2.38; 95% CI: 1.63-3.48; p < 0.001), Karnofsky index <70% (HR: 2.57; 95% CI: 1.56-4.24; p < 0.001), presence of seizures (HR: 0.55; 95% CI: 0.38-0.81; p = 0.002), IDH-wt versus IDH-m status (HR: 0.19; 95% CI: 0.11-0.33; p < 0.001), tumor location (frontal lobe vs. other sites) (HR: 0.47; 95% CI: 0.31-0.71; p < 0.001), and type of procedure (biopsy vs. surgery) (HR: 2.56; 95% CI: 1.64-3.98; p < 0.001) showed significant associations with survival.

**Table 3:** Univariate and multivariate Cox proportional hazards regression analysis for independent predictors of overall survival Variables with p < 0.05 in the univariate model were included in the multivariate analysis. Statistical significance was defined as p < 0.05 (* indicates statistical significance). HR = Hazard ratio; CI = Confidence interval; B = Regression coefficient.

Variable	Univariable Analysis Model				Multivariable Analysis Model			
	B	HR	95% CI	p-value	B	HR	95% CI	p-value
Age ≥65 years	0.87	2.38	1.63 – 3.48	< 0.001*	0.568	1.76	1.16 – 2.67	0.008*
Karnofsky <70%	0.946	2.57	1.56 – 4.24	< 0.001*	0.707	2.02	1.18 – 3.47	0.01*
Seizures	-0.582	0.55	0.38 – 0.81	0.002*	-0.72	0.93	0.61 – 1.41	0.735
IDH mutation	-1.643	0.19	0.11 – 0.33	< 0.001*	-1.37	0.25	0.14 – 0.45	< 0.001*
Frontal lobe	-0.755	0.47	0.31 – 0.71	< 0.001*	-0.48	0.61	0.39 – 0.97	0.039*
Biopsy (vs. surgery)	0.94	2.561	1.64 – 3.98	< 0.001*	0.715	2.04	1.26 – 3.31	0.004*
Male sex	-0.181	0.83	0.58 – 1.18	0.317	-	-	-	-
Weight >70 kg	-0.336	0.71	0.44 – 1.14	0.161	-	-	-	-
Smoking history	-0.084	0.91	0.51 – 1.65	0.778	-	-	-	-
Tumor size >30 mm	0.018	1.01	0.97 – 1.06	0.45	-	-	-	-
Surgery (vs. Biopsy)	-0.94	0.39	0.25 – 0.60	< 0.001*	-	-	-	-
Surgery+RT/QT (vs. others)	-0.968	0.38	0.26 – 0.54	< 0.001*	-	-	-	-

In the multivariate model, independent predictors of OS included age ≥65 years (HR: 1.76; 95% CI: 1.16-2.67; p = 0.008), Karnofsky index ≥70% (HR: 2.02; 95% CI: 1.18-3.47; p = 0.01), IDH-wt versus IDH-m status (HR: 0.25; 95% CI: 0.14-0.45; p < 0.001), tumor location (frontal lobe vs. other regions) (HR: 0.61; 95% CI: 0.39-0.97; p = 0.039), and procedure type (biopsy vs. surgery) (HR: 2.04; 95% CI: 1.26-3.31; p = 0.004). The presence of seizures lost statistical significance in the multivariate analysis (HR: 0.93; 95% CI: 0.61-1.41; p = 0.735).

## Discussion

This study represents the first national analysis of IDH mutational status in diffuse gliomas within the Panamanian healthcare system. Our findings demonstrate that the presence of an IDH mutation is significantly associated with increased OS in patients with gliomas, compared to those with IDH-wt tumors. These findings are consistent with the international literature, where IDH mutations have been recognized as a favorable prognostic biomarker in gliomas, independent of other clinical and molecular factors.

Patients with IDH-m gliomas showed markedly better survival, with median OS not reached during six years of follow-up (>73.2 months), compared with 11.4 months for the IDH-wt group. This reflects a biological advantage, evidenced by a threefold lower mortality (27.1% vs. 78.1%), suggesting that IDH mutations confer a favorable prognostic impact also in the Panamanian population.

Time-dependent survival analyses also revealed a sustained advantage in the IDH-m group, with 65.1% achieving five-year survival, compared to only 10.8% in the IDH-wt group. This difference was evident from the early months of follow-up (12 months: 81.0% vs. 48.9%; 24 months: 75.4% vs. 21.1%; 36 months: 72.4% vs. 12.3%).

Our findings are also consistent with those from the national study by Ostrom et al., which reported on more than 8,600 patients with diffuse gliomas in the National Cancer Database. In their analysis, patients with IDH-wt glioblastoma had a median survival of 17.1 months when the MGMT promoter was methylated and 12.4 months when it was unmethylated [6]. In our population, where MGMT methylation data were unavailable, the median survival for IDH-wt patients was 11.4 months (95% CI: 8.29-14.51), closely resembling the reported outcome in cases lacking MGMT methylation. This suggests that our patients likely shared a poor prognostic molecular profile.

For patients with IDH-m gliomas, the difference was even more pronounced. While Ostrom et al. reported one-year survival rates greater than 90% in grade 2 and 3 IDH-m tumors [6], our cohort showed a 12-month survival rate of 81% and a five-year survival rate of 65.1%, which are highly favorable figures given the diagnostic and therapeutic limitations of our setting.

Sarac et al. reported significantly longer median OS in IDH-m glioblastomas (16.0 months; 95% CI: 11.41-20.58) compared with IDH-wt tumors (6.5 months; 95% CI: 0.0-15.35; p = 0.030). Survival differences were also evident at six months (92.8% vs. 49.0%) and 1 year (69.2% vs. 2.5%) [7]. Similarly, Aboubakr et al., in a retrospective review of 976 IDH-wt glioblastomas, reported a median survival of 11.2 months, comparable to our finding of 10.6 months (95% CI: 8.42-12.78). These results underscore the inherent aggressiveness of IDH-wt glioblastomas and the rarity of long-term survivors [8].

A meta-analysis by Xia et al., including 55 studies and 9,487 patients, demonstrated a significant survival advantage in gliomas with IDH1/2 mutations: OS (HR = 0.39; 95% CI: 0.34-0.45; p < 0.001) and progression-free survival (PFS) (HR = 0.42; 95% CI: 0.35-0.51; p < 0.001) [9]. Similarly, Zou et al., analyzing 12 studies with 2,190 patients, reported pooled HRs of 0.33 (95% CI: 0.25-0.42) for OS and 0.38 (95% CI: 0.21-0.68) for PFS, indicating a 67% and 62% reduction in mortality and tumor progression, respectively. These results align with our findings, where IDH mutations conferred a 75% lower risk of death (HR = 0.25; 95% CI: 0.14-0.45; p < 0.001) [5].

In particular, younger patients, those with better functional status (Karnofsky ≥70%), tumors located in the frontal lobe, and those who underwent surgical resection rather than biopsy had a more favorable prognosis. Additionally, the presence of seizures at diagnosis was associated with improved survival. The Karnofsky index is a well-known predictor of glioma outcomes, as higher scores are linked to better prognosis and increased tolerance to multimodal treatments [10].

The increased survival observed in patients presenting with seizures at diagnosis may be explained by studies suggesting that D-2-hydroxyglutarate (D-2HG) increases neuronal activity, thereby promoting epileptogenesis in IDH-m gliomas. This enhanced neuronal activity is associated with metabolic alterations and activation of the mTOR signaling pathway, suggesting that D-2HG contributes to seizure onset and may be related to less aggressive tumor behavior and improved survival [11].

The combination of surgery with adjuvant RT and temozolomide-based CT significantly improved survival, underscoring the importance of multimodal management in glioma patients. Cohen et al. reported that IDH-m tumors respond better to this combined approach, which may partly explain the superior outcomes observed in our cohort [4]. Similarly, Hart et al., in a meta-analysis of three randomized trials, showed that temozolomide administered concurrently and adjuvantly with RT prolonged survival and delayed progression compared with RT alone, without compromising quality of life, despite an increase in early adverse effects [12].

Moreover, the relationship between IDH status and tumor location may offer additional insights for surgical planning. IDH-m gliomas tend to be located in brain regions more amenable to extensive resection, which may contribute to longer survival [13]. This should be taken into consideration when designing surgical strategies, potentially favoring more aggressive resections in patients with favorable prognoses [13].

Regarding the study's limitations, its retrospective design entails certain inherent challenges, including reliance on the quality of medical records and potential biases in data collection. Variables such as heterogeneity in treatment regimens and the absence of additional biomarkers may have influenced the observed outcomes.

Given the impact of IDH status on survival, future research in Panama and across Latin America should focus on evaluating the effect of specific treatment strategies based on tumor molecular profiles. Furthermore, prospective studies incorporating additional biomarkers, such as MGMT promoter methylation, TERT promoter mutations, CDKN2A/B homozygous deletion, and ATRX, would allow for more precise prognostic and predictive assessments. Evaluation of IDH1 inhibitors may also represent a promising therapeutic avenue, given the growing interest in targeted therapies for this mutation. Additional studies are needed to determine the effectiveness of these agents in combination with current standard treatments and their long-term impact on survival.

## Conclusions

This retrospective Panamanian study underscores the prognostic relevance of IDH mutation status in glioma patients in a Latin American setting. IDH mutations were strongly associated with improved OS, even after statistical adjustments for age, functional status, tumor location, and therapeutic modalities. These findings reaffirm the importance of IDH status as a key biomarker and emphasize the need to expand access to molecular diagnostics in resource-limited environments. Incorporating IDH testing into routine clinical practice and designing region-specific prospective studies will be essential to guide therapeutic decisions and improve outcomes for glioma patients in LMICs.
